# Recurrent Water Level Fluctuation Alleviates the Effects of Submergence Stress on the Invasive Riparian Plant *Alternanthera philoxeroides*

**DOI:** 10.1371/journal.pone.0129549

**Published:** 2015-06-12

**Authors:** Haijie Zhang, Renqing Wang, Xiao Wang, Ning Du, Xiuli Ge, Yuanda Du, Jian Liu

**Affiliations:** 1 Institute of Environmental Research, Shandong University, Jinan, China; 2 School of Life Sciences, Shandong University, Jinan, China; 3 Shandong Provincial Engineering and Technology Research Center for Vegetation Ecology, Shandong University, Jinan, China; 4 School of Environment Science and Engineering, Qilu University Technology, Jinan, China; University of Vigo, SPAIN

## Abstract

Recurrent water level fluctuation and submergence of plants are common in riparian zones. Our study objectives were to test the independent and interactive effects of submergence level and fluctuation frequency on a globally important riparian invasive plant, *Alternanthera philoxeroides*. To this end, we conducted a greenhouse experiment, in which ramets of the plants, obtained from a wetland in China, were treated with four fluctuation frequencies (0, 3, 6, and 12 cycles over a 96-day experimental period) under three water levels (0, 10, and 30 cm). We found that effects of fluctuation frequency were non-significant, negative, and positive under water levels of 0, 10 and 30 cm, respectively. As fluctuation frequency increased, the effects of increasing water level decreased significantly. When water levels were high, *A*. *philoxeroides* allocated greater biomass to shoot production probably in order to elongate and escape from submergence. However, as fluctuation frequency increased, biomass investment in roots and leaves also increased, probably in order to maximize nutrient absorption and photosynthesis, respectively. These results suggest that water level fluctuation may alleviate the effects of submergence on *A*. *philoxeroides*. In addition, *A*. *philoxeroides* showed significant phenotypic plasticity, adjusting its functional traits, such as number of nodes and leaves per stem, as well as stem diameter and pith cavity diameter, according to recurrent water level fluctuation. We conclude that *A*. *philoxeroides* may perform better in shallow water zones under conditions of disturbance that include recurrent water level fluctuation. This ability to adapt to disturbance likely promotes its growth and invasion in disturbed habitats.

## Introduction

Fluctuating resources and stress are common in various kinds of habitats and have profound consequences for individuals, populations, communities and ecosystems [1–3]. In wetlands, water level fluctuation occurs frequently due to climate change, and human disturbance [4–11]. While rising water levels submerge plants, falling water levels indicate water scarcity, creating water stress for plants. Many experiments have been performed to study the effects of submergence on riparian plants, reporting various morphological and physiological adjustments to submergence [12–18]. Other studies have addressed the response of plants to submergence and de-submergence [9, 19], as well as the effects of the amplitude of water level fluctuation [5, 20–23]. Nevertheless, recurrent water level fluctuation occurs as a result of extreme weather, such as sudden, heavy rainstorms, and artificial disturbances, such as the construction of dams, which control the release of water into rivers. In the face of increasing submergence and water level fluctuation, research on the effects of such ‘recurrence’ on plants in riparian zones is critical. Yet, despite substantial research on the effects of water level fluctuations on riparian and aquatic plants [5, 14, 17, 23], few studies explore the role of recurrent water fluctuation in influencing their growth.

*Alternanthera philoxeroides* (Mart.) Griseb is an invasive perennial herb that has colonized riparian areas in many parts of the world [19, 24–26]. Its invasion success appears to be driven mainly by its vegetative propagation and high phenotypic plasticity [24, 25]. *A*. *philoxeroides* can survive and grow when submerged [27], and is better adapted to submergence than its non-invasive congener *Alternanthera sessilis* [28]. It is also able to recover effectively after de-submergence [9, 19, 29, 30]. However, little is known about the effects of recurrence on this invasive plant, particularly at varying water levels. So the plant is an ideal material for studying the effects of recurrent water level fluctuation on riparian plants.

In order to test the effects of recurrent water level fluctuation on *A*. *philoxeroides* at varying depths of submergence, we conducted a greenhouse experiment in which we measured changes in biomass accumulation and plant functional traits in *A*. *philoxeroides* at three water levels and four fluctuation frequencies. Our study objectives were to answer the following questions: (1) Does water level fluctuation alter the effects of water level increase on the growth of *A*. *philoxeroides*? We predicted that the water level fluctuation would alleviate the effects of water level increase on the plant growth because the water level fluctuation might provide opportunity for the plant to adapt to the submergence. (2) How do the functional traits of *A*. *philoxeroides* respond to water level fluctuation? We predicted that the functional traits of the plant might respond to the changing environment through phenotypic plasticity.

## Materials and Methods

### Ethics statement

We collected field samples for our study with the official permission of the Environmental Protection Bureau of Weishan County, and the Management Committee of the Xinxue River constructed wetland. We did not collect any endangered or protected plants, or any animal species.

### Data collection

We collected *A*. *philoxeroides* samples from Xinxue River constructed wetland in northern China (34°45.978′N, 117°8.936′E). In early May 2014, we collected plant stolons, with leaves, from the wetland and cultured them in a nursery for the greenhouse experiments. In China, there are low levels of genetic diversity in *A*. *philoxeroides* and it spreads by vegetative propagation [25]. We collected plant stolons in only one spot and believed all plant stolons belonged to one genotype. We conducted our experiments in the greenhouse of the Fanggan Research Station of Shandong University, China. During the experiment, the average temperature in daytime and night varied from 20.3 to 36.2°C and from 10.0 to 26.7°C, respectively. The relative humidity ranged from 30.9 to 88.0%.

On June 7, 2014, we collected similar *A*. *philoxeroides* ramets (5 cm from apex to breakpoint, with four leaves), which we planted in pots (14 cm × 16 cm, diameter × height) filled with 7.5 kg of a mixture of loam and sand (w/w = 3:1). This substrate contained 50.20 mg/kg, and 31.14 mg/kg of nitrogen (N), and phosphorus (P) concentrations, respectively. After two weeks of recovery growth acclimatization (without submergence), we selected seventy-eight similar ramets in internode number (3), leaf number (6), and stem length (7.50 ± 0.62 cm) for our experiments; in six of these ramets, we measured initial biomass (0.1239 ± 0.0235 g).

We examined the effects of three water levels and four fluctuation frequencies on the remaining 72 experimental ramets. Field observations suggest that *A*. *philoxeroides* prefers shallow water in wetlands, and a preliminary test revealed that individuals that belong to the same size class as our experimental ramets had very low survival under water at depths of 50 cm or more. Therefore, we selected three water levels—0 cm, 10 cm, and 30 cm (labeled as WL0, WL10, and WL30 respectively)—lower than this ‘critical’ level of 50 cm. We set different water level through putting the pots into tanks (48 cm × 60 cm, diameter × height) filled with different water volume.

We also subjected the ramets to four different frequencies of water level fluctuation. Each fluctuation cycle consisted of a fixed period of time under high water level (10 cm higher than the average level set for the group) and an equal period under low water level (10 cm lower than the group average); each group had one of three water levels (as described above). We controlled water level in tanks to achieve change of water level for pots. All treatments were initiated with their high water levels. Our four fluctuation treatments (FF1, FF2, FF3, and FF4) consisted of twelve, six, three, and zero fluctuation cycles, respectively, over the course of the 96-day experiment (Table 1) [31]. FF4 represented constant water levels with no fluctuation. In total, we had 12 factorial combinations (groups), each containing six randomly assigned replicate ramets (n = 6). We renewed the water in the pots every eight days and water was added daily to offset evaporation.

**Table 1:** Number of days of exposure of *Alternanthera philoxeroides* ramets under different fluctuation treatments (FF1, FF2, FF3 and FF4) with their respective cycles of fluctuations (0, 3, 6 and 12 cycles) over a 96-day experimental growth period, for any particular level of submergence.

Fluctuation frequency	Number of fluctuation cycles	Days at high water level (one cycle)	Days at low water level (one cycle)
FF1	12	4	4
FF2	6	8	8
FF3	3	16	16
FF4	0	—	—

### Measurements and calculation

At the end of the 96-day experimental period, we measured the morphological characteristics—number of branches, nodes, and leaves per stem, as well as maximum stem diameter, and stem wall thickness—of the ramets. Then, we harvested and separated each ramet into storage roots (root diameter ≥ 1 mm), fine roots (root diameter < 1 mm) [25], stems and leaves. We dried the sorted plants at 80°C for 48 h after which we measured their biomass. We calculated total biomass (a measure of biomass accumulation by the plant) as the sum of the biomass values of each of the plant parts (storage roots, fine roots, stems, and leaves). We calculated the mass ratios of roots, stems, and leaves as the ratio of the biomass of each of those parts to total biomass, e. g. root mass ratio = (storage root biomass + fine root biomass)/ total biomass. Each ratio represented the percent allocation of total biomass to that particular plant part. Finally, we calculated storage root to fine root ratio, pith cavity diameter, and specific leaf area (SLA) as: Storage root to fine root ratio = storage root biomass/fine root biomass, Pith cavity diameter = maximum of stem diameter—stem wall thickness × 2, Specific leaf area = leaf area/leaf dry mass.

### Statistical analyses

At the beginning of statistical analyses, the normality and homogeneity of variance of data were checked. To fulfill the model assumptions, sqrt- transformation was applied in storage root biomass and ln-transformation was applied in root biomass, fine root biomass, node number, leaf number and maximum of stem diameter. Then we used two-way analysis of variance (ANOVA) with post-hoc Duncan’s tests (with *p* = 0.05) to assess the significance of the effects of water level and fluctuation frequency on plant response variables (n = 72). In cases where there were significant interactions between water level and fluctuation frequency, in their effects on plant responses, we used one-way ANOVAs to determine these interactive effects. We used SPSS 18.0 (SPSS Inc., Chicago, USA) to conduct statistical analyses, and Origin Pro 8.0 (Originlab Co., Northampton, MA) to prepare figures.

## Results

### Biomass accumulation and allocation

**Fig 1:**
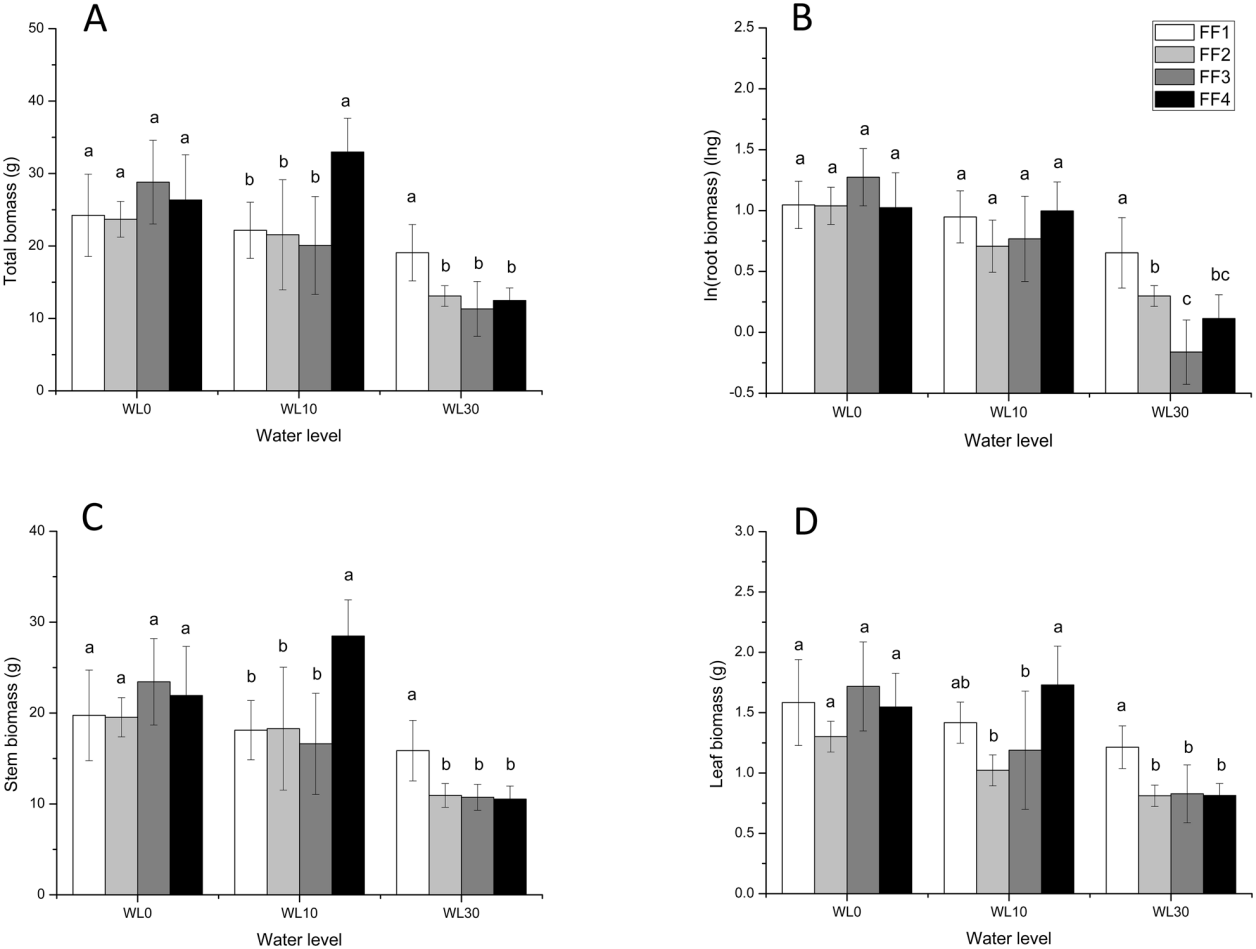
Effects of fluctuation frequency at different water levels on various biomass variables in *A*. *philoxeroides*. A: Total biomass, B: ln(root biomass), C: Stem biomass, D: Leaf biomass (D). Different letters at each water level denote significant differences (*p* ≤ 0.05) with Duncan’s test.

Water level and fluctuation frequency had significant interactive effects on biomass accumulation, measured as total biomass (Table 2). Analyses of the two factors, separately, revealed that at a water level of 0 cm, total biomass did not vary across fluctuation frequency (F = 1.181, *p* > 0.05, Fig 1A). However, at a water level of 10 cm, total biomass at FF4 (constant water level) was significantly higher than at the other three fluctuation frequencies (FF1, FF2 and FF3; F = 6.013, *p* < 0.01), Finally, at the highest water level (30 cm) total biomass was significantly higher for plants exposed to the highest fluctuation frequency (FF1, 12 cycles of fluctuation) than for those exposed to the other three frequencies (6, 3, and 0 cycles; F = 7.140, *p* < 0.01). The effects of fluctuation frequency, at each water level, on root, stem, and leaf biomass were similar to those on total biomass (Fig 1).

**Table 2:** Results of the two-way ANOVA comparing the response of biomass accumulation and allocation, as well as plant functional traits in *A*. *philoxeroides*, to varying water levels (WL) and fluctuation frequencies (FF).

Measurement indices	F-value and its significance		
	WL	FF	WL * FF
**Biomass accumulation and allocation**			
Total biomass	32.749***	2.464ns	4.354**
ln(root biomass)	70.407***	3.759*	5.346***
Stem biomass	27.278***	2.576^ns^	4.443**
Leaf biomass	27.124***	5.769**	2.988*
Root mass ratio	12.869***	4.827**	1.198^ns^
Stem mass ratio	7.798**	7.582***	1.374^ns^
Leaf mass ratio	8.657**	4.787**	1.828^ns^
**Root biomass accumulation and allocation**			
Sqrt(storage root biomass)	34.349***	0.661^ns^	2.211^ns^
ln(fine root biomass)	35.357***	4.686**	3.290**
Storage root to fine root ratio	13.331***	1.339^ns^	1.151^ns^
**Plant functional traits**			
ln(node number)	23.655***	0.978^ns^	4.820***
Branch number	0.159^ns^	1.981^ns^	1.639^ns^
ln(maximum of stem diameter)	15.086***	4.921**	5.817***
Pith cavity diameter	4.794*	6.731**	4.833***
Stem wall thickness	13.739***	9.406***	1.702^ns^
ln(leaf number)	13.805***	2.474^ns^	2.267^ns^
Specific leaf area	12.338***	2.112^ns^	0.961^ns^

There were six replicates in each treatment (n = 6). Asterisks and “ns” indicated significant effects:

*: *p* ≤ 0.05,

**: *p* ≤ 0.01,

***: *p* ≤ 0.001, and ns: *p* > 0.05.

**Fig 2:**
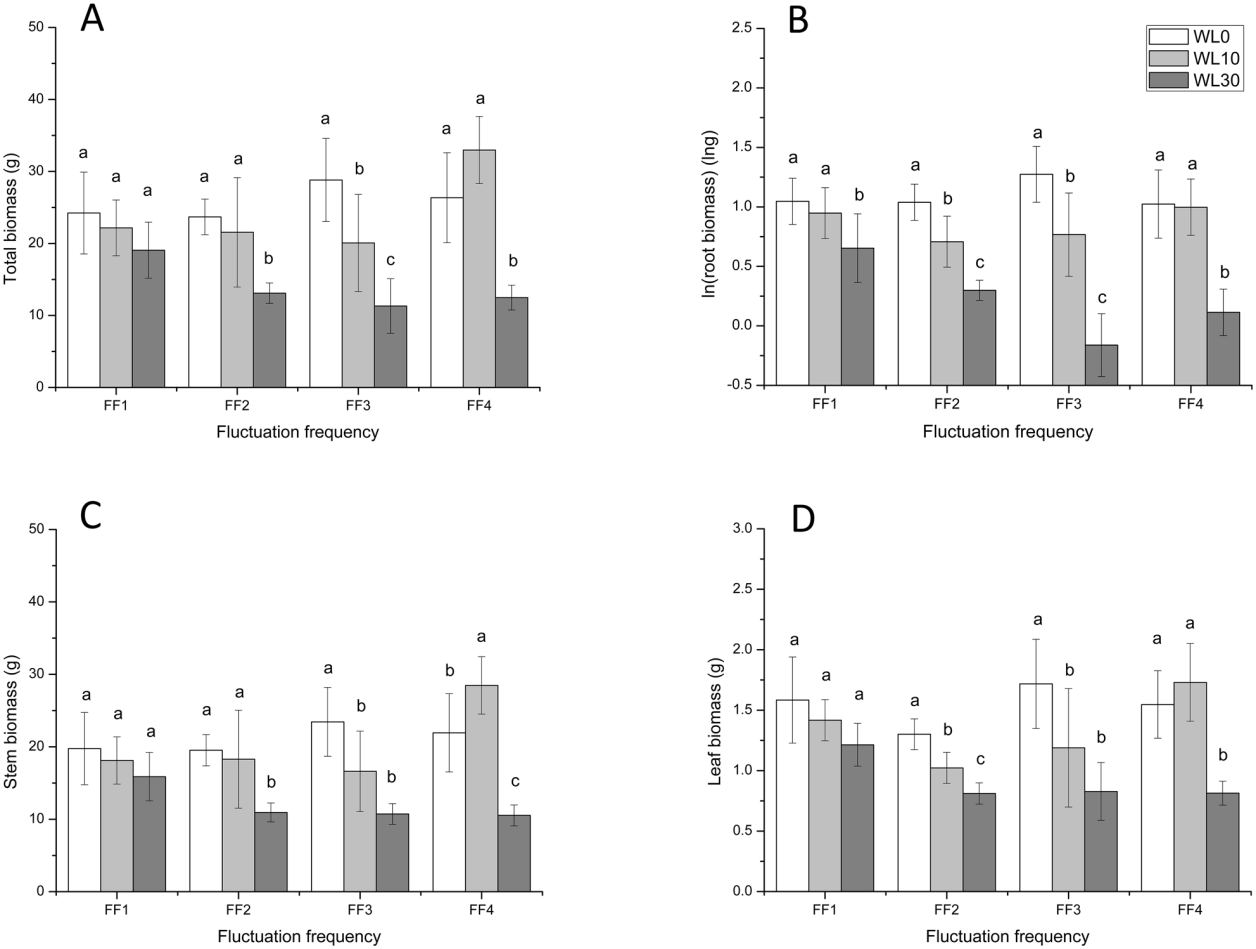
Effects of water level at different fluctuation frequencies on biomass accumulation in *A*. *philoxeroides*. A: Total biomass, B: ln(root biomass), C: Stem biomass, D: Leaf biomass. Different letters at each kind of fluctuation frequency denote significant differences (*p* ≤ 0.05) with Duncan’s test.

Total biomass at different water levels was not significantly different at the highest fluctuation frequency (12 cycles; F = 1.945, *p* > 0.05). However, as fluctuation frequency decreased, the effect of water level became visible (FF2 (F = 8.560, *p* < 0.01) and FF3 (F = 12.979, *p* < 0.01)). When fluctuation frequency reduced to zero (constant water level), total biomass at WL0 (no submergence) was similar to that under 10 cm of water, and much higher than that under 30 cm (F = 16.322, *p* < 0.001). The responses of root, stem, and leaf biomass were similar to that of total biomass (Fig 2).

**Fig 3:**
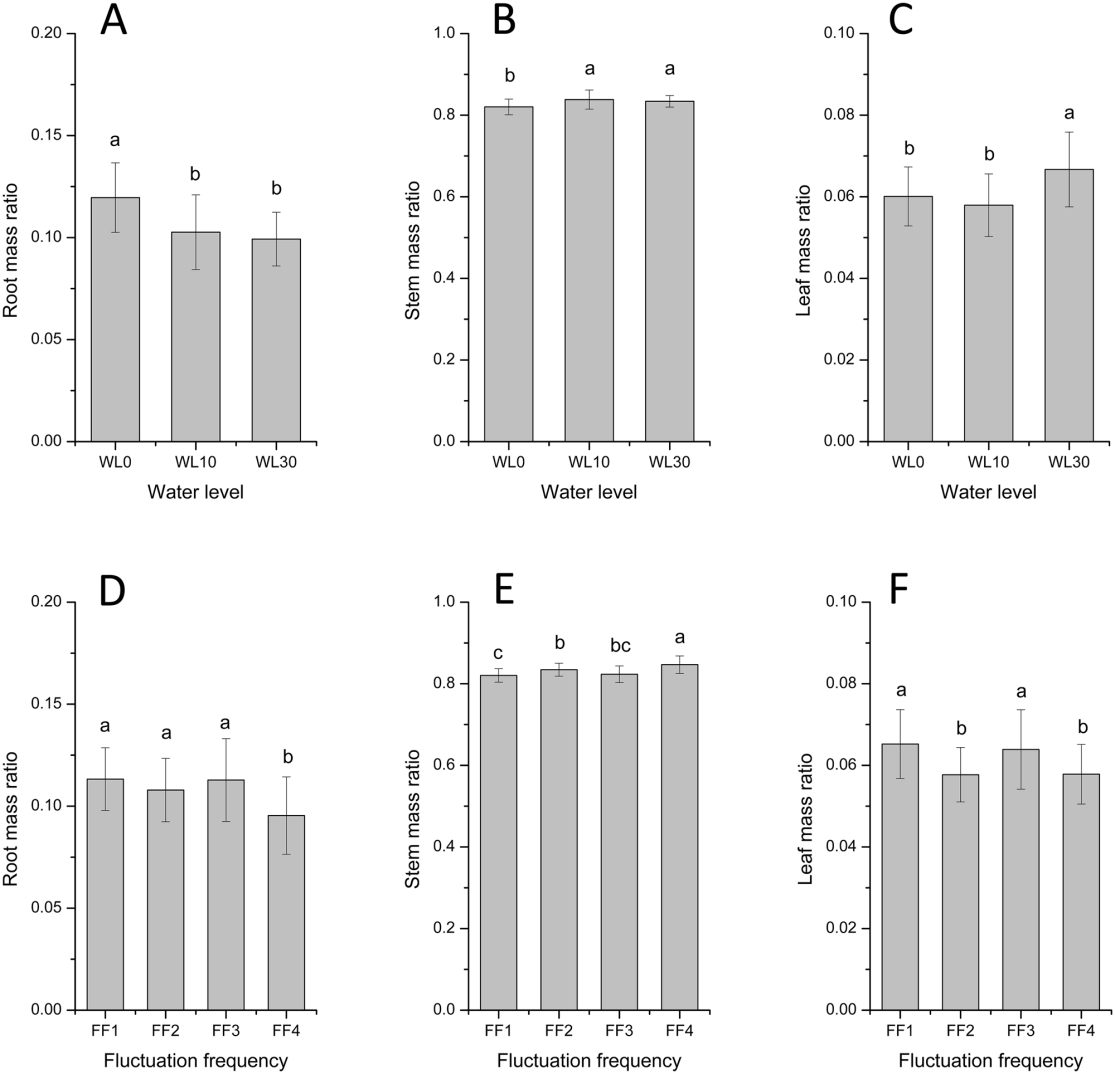
Biomass allocation in *A*. *philoxeroides* under different water levels and fluctuation frequencies. Root mass ratio (A and D), Stem mass ratio (B and E) and Leaf mass ratio (C and F). Different letters denote significant differences (*p* ≤ 0.05) with Duncan’s test.

There were no interactive effects between water level and fluctuation frequency on biomass allocation (Table 2). Water level had significant but diverse effects on root, stem, and leaf mass ratios (F = 12.689, 7.798, 8.657 and *p* < 0.001, < 0.01, < 0.01, respectively). Water level had a negative effect on root mass ratio (biomass allocation to roots was higher under no submergence than under 10 and 30 cm of water; Fig 3A). However, it had a positive effect on both stem and leaf mass ratio, both of which increased as water level increased from 0 to 30 cm (Fig 3B and 3C, respectively). The influence of fluctuation frequency on biomass allocation also varied among root, stem and leaf (Table 2). Root and leaf mass ratios (Fig 3D and 3F respectively) were significantly higher when water levels fluctuated (FF1, FF2, and FF3) than in still water (FF4), whereas stem mass ratio (Fig 3E) decreased as fluctuation frequency increased.

### Root biomass accumulation and allocation

**Fig 4:**
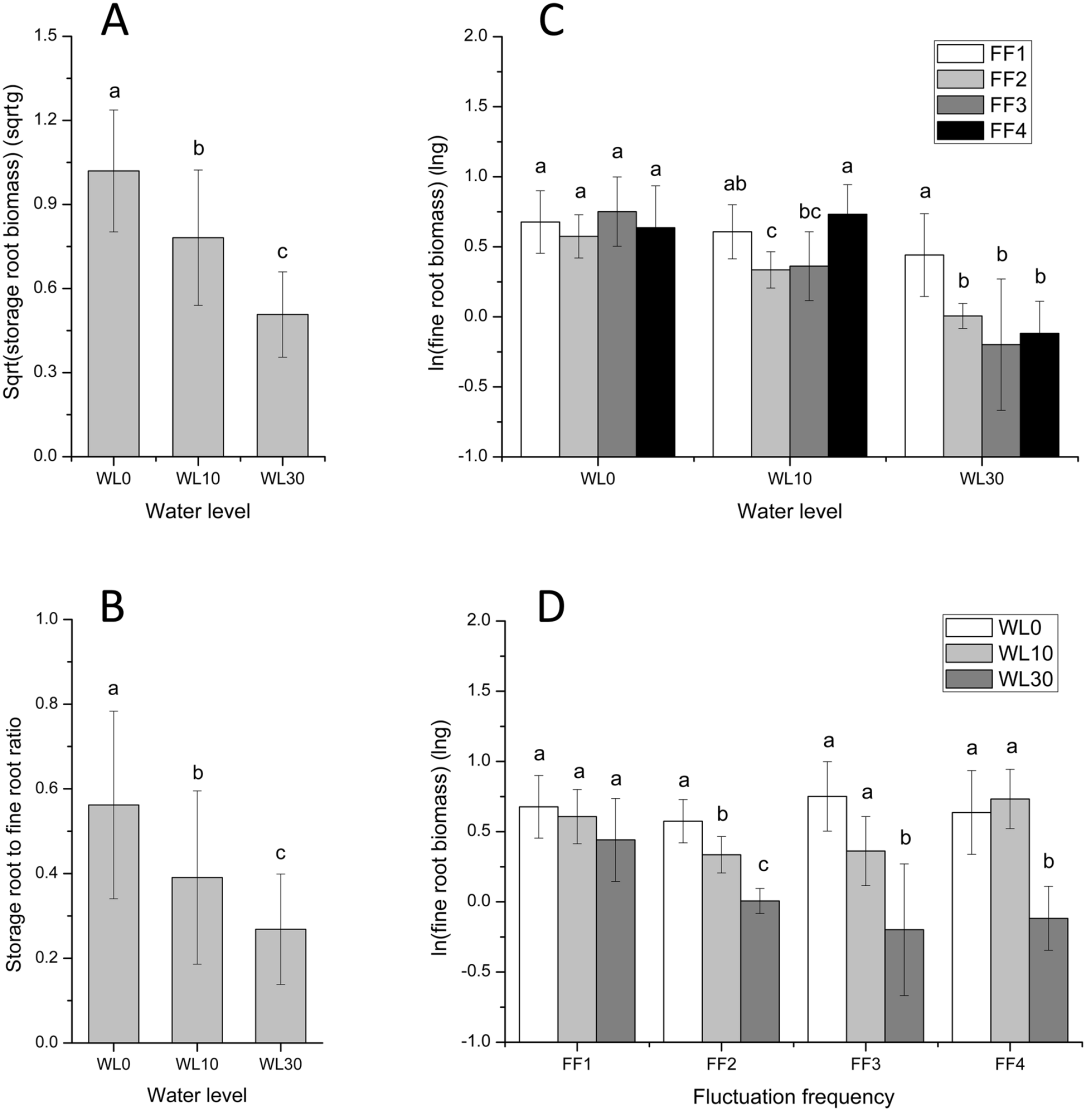
Root biomass accumulation and allocation in *A*. *philoxeroides* under different water levels and fluctuation frequencies. Sqrt(storage root biomass) (A), Storage root to fine root ratio (B), ln(fine root biomass) (C and D). Different letters denote significant differences (*p* ≤ 0.05) with Duncan’s test (C at each water level, D at each fluctuation frequency).

Both storage root biomass and storage root to fine root ratio were negatively influenced by water level (Fig 4A and 4B) but were not influenced by fluctuation frequency (Table 2). On the other hand, fine root biomass was affected by water level, fluctuation frequency, and the interaction between them (Table 2; Fig 4C and 4D). At 0 cm of submergence, fine root biomass was not affected by fluctuation frequency (F = 0.631, *p* > 0.05). However, as water levels rose, fluctuation frequency had significant effects on fine root biomass. At 10 cm of submergence fine root biomass was highest when there was no water level fluctuation (FF4; F = 5.182, *p* < 0.05); however, under 30 cm, fine root biomass was highest under the highest fluctuation frequency (12 cycles; F = 4.810, *p* < 0.05; Fig 4D).

The effects of water level on fine root biomass, at different fluctuation frequencies, were similar to those on total biomass. Under high fluctuation frequency (12 cycles), water level had no effect, but as fluctuation intensity decreased (from 12 to 3 cycles), high water levels strongly inhibited the growth of fine roots. Finally, when water fluctuation ceased, fine root biomass at no (0 cm of water), and low (10 cm) submergence was higher than that at high submergence (30 cm; Fig 4E).

### Plant functional traits

**Fig 5:**
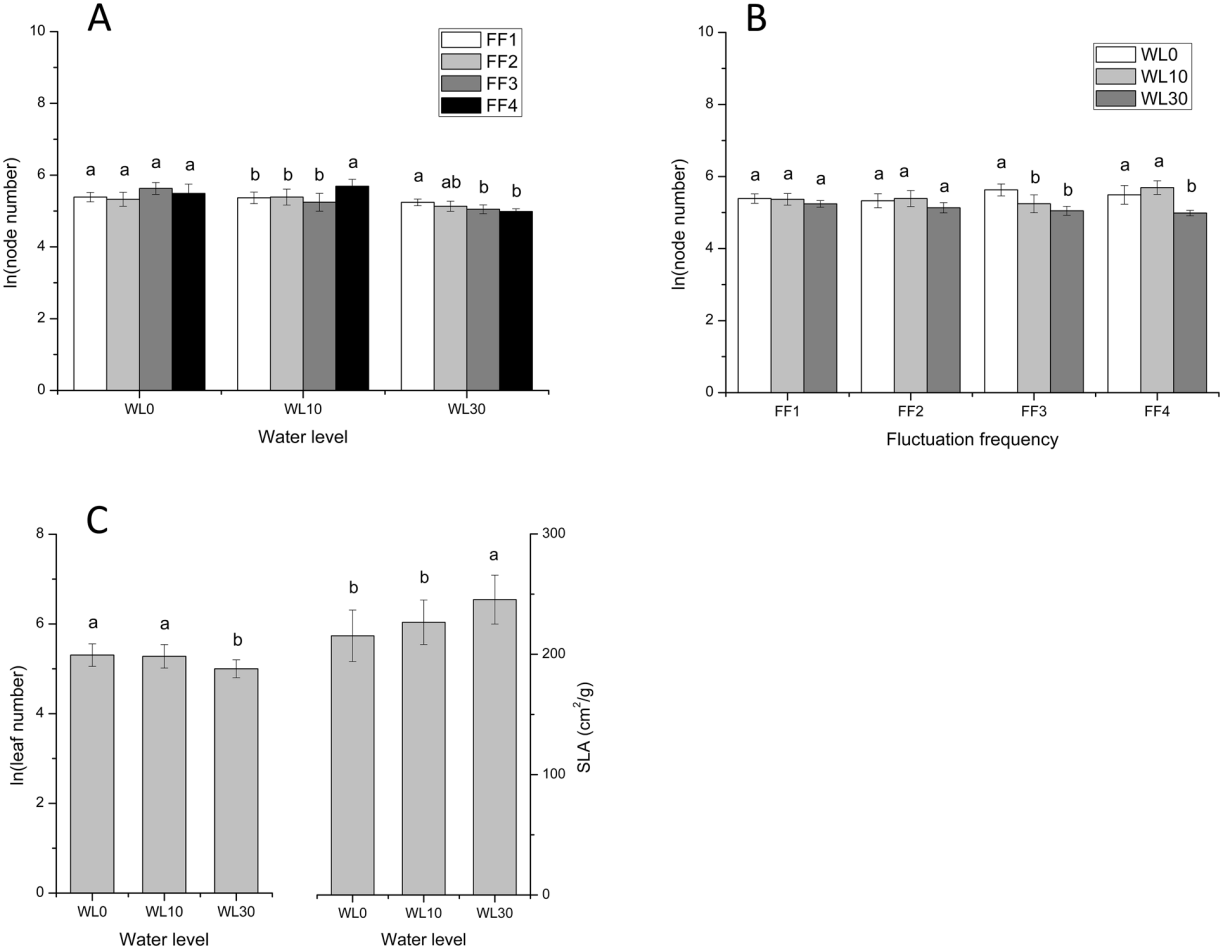
Effects of water level and fluctuation frequency on plant functional traits in *A*. *philoxeroides*. ln(node number), ln(leaf number) and SLA of *A*. *philoxeroides* at different water levels and fluctuation frequencies. Different letters denote significant differences (*p* ≤ 0.05) with Duncan’s test (A at each water level and B at each fluctuation frequency).

Our analyses of the effects of water level and fluctuation frequency, on various morphological characteristics of stems and leaves, revealed that water level influenced the number of nodes (node number) on stems but not the number of branches (branch number; Table 2). However, the effects of fluctuation frequency on node number, at water levels of 0, 10, and 30 cm, were non-significant, negative, and positive, respectively (Fig 5A). Furthermore, the differences between stress of increasing water levels on node number became more obvious from FF1 (12 cycles) to FF4 (still water; Fig 5B). Leaf number (number of leaves per stem) and specific leaf area (SLA) were influenced only by water level and not fluctuation frequency (Table 2). At high water levels, plants had fewer leaves and higher SLA (Fig 5C).

**Fig 6:**
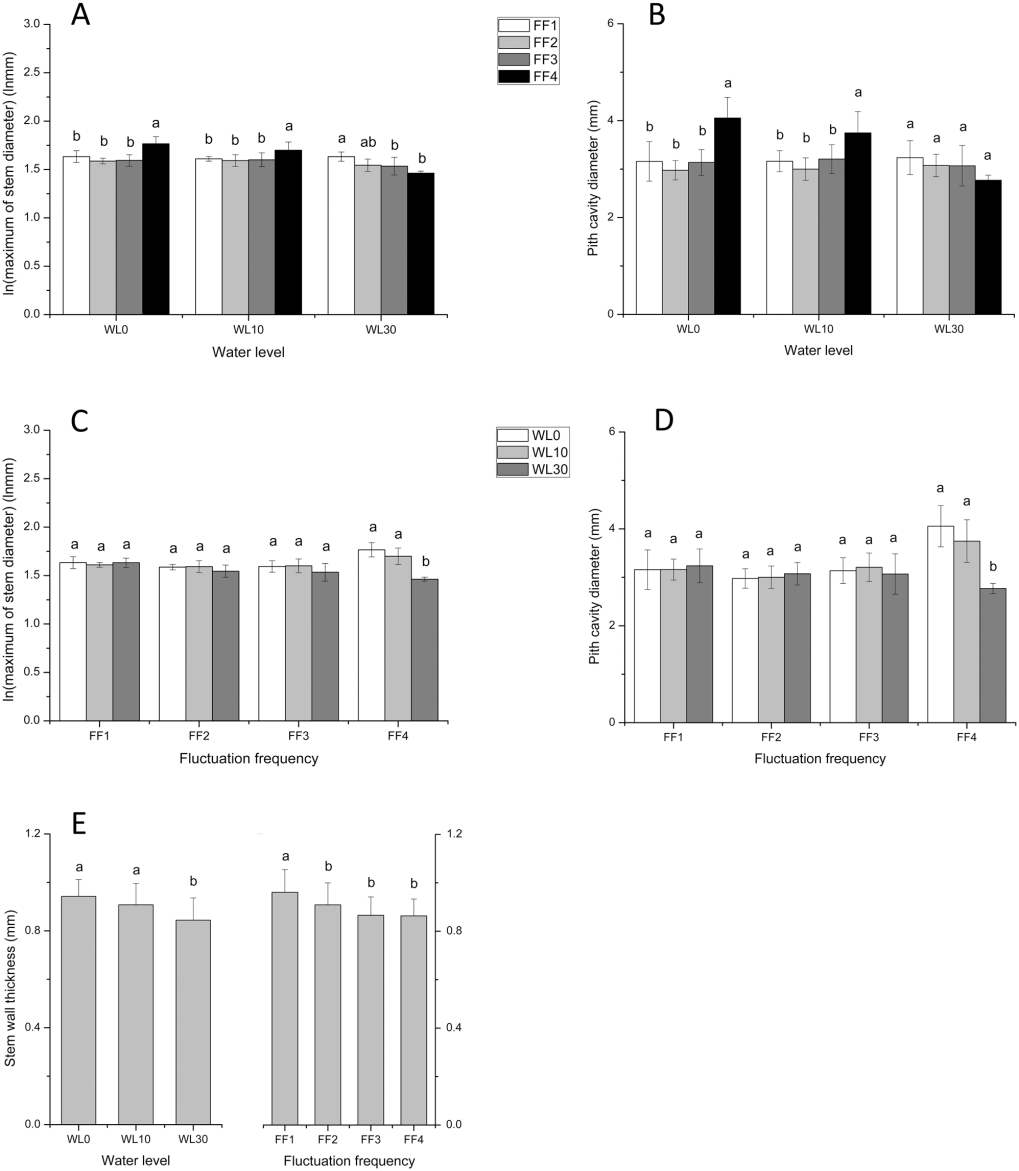
Effects of water level and fluctuation frequency on stem functional traits in *A*. *philoxeroides*. ln(maximum of stem diameter) (A and C), Pith cavity diameter (B and D) and Stem wall thickness (E). Different letters denote significant differences (*p* ≤ 0.05) with Duncan’s test (A and B at each water level, C and D at each fluctuation frequency).

Stem diameter varied across the different experimental water levels but did not respond to fluctuation (Fig 6). At all levels of fluctuation (12, 6, and 3 cycles), maximum stem diameter and pith cavity diameter were smaller under no, and low submergence (10 cm of water) but larger under high submergence (30 cm) than those under still water (Fig 6A and 6B). Conversely, when there was no fluctuation (still water), increasing water levels resulted in significant decreases in maximum stem diameter and pith cavity diameter (F = 14.375, *p* < 0.01 and F = 10.612, *p* < 0.01; Fig 6C and 6D). Stem wall thickness was negatively influenced by water level but positively influenced by fluctuation frequency (Fig 6E).

## Discussion

Just like our prediction above, recurrent water level fluctuation promoted biomass accumulation and alleviated the stress effects of submergence on *A*. *philoxeroides*. In addition, the functional traits of the plant responded to water level fluctuation significantly.

### Interactive effects between water level and fluctuation frequency

By comparing biomass accumulation (total biomass) under different fluctuation frequencies for each water level, we found that while at 0 cm of water, variation in fluctuation frequency did not affect biomass accumulation. At both higher water levels (10 cm and 30 cm), fluctuation had important but opposite effects on the growth of *A*. *philoxeroides*. Under 10 cm of water, plants fared much better with no fluctuation; however, under 30 cm of water, intense water level fluctuation (12 cycles) promoted plant biomass accumulation. Water level did not significantly affected biomass accumulation at FF1 and the difference of biomass accumulation between different water levels became wider from FF2 to FF4 (Fig 2). In other words, lower rates of fluctuation benefit *A*. *philoxeroides* when it is in shallow water or not submerged, but in deep water, more frequent fluctuation is more beneficial than little or no fluctuation.

Although it is known that submergence (increasing water levels) can inhibit plant growth [4, 12, 15, 27, 32], our study shows that the effects of water level are dependent on fluctuation frequency. Plants that are able to escape levels of submergence that are disadvantageous to their growth can continue to accumulate biomass. In the case of *A*. *philoxeroides*, inhibition of growth is likely mediated by decreases in gas diffusion (CO_2_ for photosynthesis and O_2_ for respiration) under submergence [4, 33]. The patterns we report here suggest that high frequencies of water level fluctuation (such as 12 cycles over the growth period) ‘rescue’ *A*. *philoxeroides* from constant submergence in deep water, increase gas diffusion [5], and, thereby, enable it to continue to grow even during times of flooding and under submergence-related stress. In contrast, while shallow water is conducive for *A*. *philoxeroides* growth [24], at low levels of submergence, fluctuation can actually inhibit plant growth. Our results are in contrast with those of a similar study on *Ottelia alismoides* [20] but similar to those of the study about *Acorus calamus* [34]. A submerged macrophyte, *O*. *alismoides* did not respond to changes in fluctuation frequency [20], unlike *A*. *philoxeroides* and *A*. *calamus*, two emergent macrophytes, which both take “escape” strategy to cope with submergence [29, 34]. Therefore, we can conclude that stressing effects of submergence on *A*. *philoxeroides* will be alleviated or removed by recurrent water level fluctuation [34].

Water level and fluctuation frequency exerted independent effects on biomass allocation in *A*. *philoxeroides*. Increasing submergence decreased investment in roots, but increased investment in stems and leaves, suggesting that under submergence, *A*. *philoxeroides* likely invests in greater biomass allocation to aboveground parts. This conclusion was also reported in *Spartina maritima* since they had taller shoot at more submerged locations [35]. “Escape” responses to submergence by shoot elongation have been reported for *A*. *philoxeroides* [4, 29] and *Phragmites australis* [36]. Conversely, higher fluctuation frequency increased biomass investment in roots and leaves, but decreased investment in stems, indicating that water level fluctuation increases the time spent by the plant outside water, and thereby mitigates submergence stress on *A*. *philoxeroides*. Moreover, rapid shoot elongation during submergence may be adverse to plant growth after de-submergence (when water levels fall) [37], and “escape” strategy is adopted by *A*. *philoxeroides* under the force of submergence. Compared with stable water level, the trade-off made by *A*. *philoxeroides* between submergence and shoot elongation is lower under the conditions of recurrent water level fluctuation. Patterns of biomass accumulation and allocation, reported here, indicate that water level fluctuation alleviates submergence stress on *A*. *philoxeroides*, contributing to plant growth under submergence.

When submergence occurs, roots are the first to suffer from the stress and respond to it [38]. Several flooding-tolerant plants, such as *Cotula coronopifolia*, *Meionectes brownii* and *Oryza sativa*, produce adventitious roots in order to overcome submergence [38–40]. Although *A*. *philoxeroides* also produced fine roots in response to submergence, the growth of these roots varied across water level and fluctuation frequency. Our finding—fine roots did not respond to submergence under frequent water level fluctuation and when fluctuation decreased or ceased, submergence inhibited fine root growth—are in accordance with root traits under submergence [33]. Increasing water levels increased the allocation of biomass to the production of fine roots, suggesting that *A*. *philoxeroides* produces fine roots as an adaptation to submergence [38]. Besides, effects of water level and fluctuation frequency on fine root biomass were similar to total biomass. Thus, water level fluctuation might affect the overall growth of *A*. *philoxeroides* by promoting the production of fine roots.

In addition, *A*. *philoxeroides* produced storage roots which could regenerate new ramets in the next year [25, 41]. In this study, storage root biomass and storage root to fine root ratio decreased when water level increased. Terrestrial *A*. *philoxeroides* allocates more biomass to storage roots than its aquatic counterpart [25]. On the other hand, the investment of greater energy by aquatic *A*. *philoxeroides* in producing stems, for the formation of independent free-floating mats [24], explains the negative relationship between storage root biomass (and storage root to fine root ratio) and water level. However, storage roots were not affected by fluctuation frequency, suggesting that water level fluctuation might not influence the production of new shoots in next spring. They are, however, influenced by water level.

### Effects of water level fluctuation on plant functional traits

The varied responses of node number to the different rates of water level fluctuation (non-significant, negative, and positive under 0, 10, and 30 cm of water, respectively), are consistent with the effects of water level and fluctuation frequency on stem biomass in plant traits. The absence of any influence of fluctuation frequency on leaf traits, such as leaf number and SLA, in *A*. *philoxeroides*, is different from those of *A*. *calamus* [34]. This may be because leaves of *A*. *philoxeroides* soon escaped to water surface while *A*. *calamus* is leaves basal, which blades were still submerged during water level fluctuation. Similarly, the range of responses exhibited by stem diameter, pith cavity diameter, and stem wall thickness, to various levels of submergence, demonstrate phenotypic plasticity in *A*. *philoxeroides* [42], as a response to submergence-related stress. Specifically, at stable water levels (no fluctuation) larger stem diameters and pith cavity diameters can increase plant buoyancy, enabling the plant to float on the water surface, and facilitating gas exchange [33]; thinner walls are beneficial to gas exchange under water as well [42]. Furthermore, the increase in stem wall thickness, observed under conditions of fluctuation, appears to be a defense against the adverse effects of frequent water level fluctuation. Thus, we report patterns that strongly support the view that *A*. *philoxeroides* exhibits great plasticity to changing environments [25, 43, 44], including flooding regimes in their riparian wetland habitats.

## Conclusions

In *A*. *philoxeroides*, submergence stress, which is known to hamper growth [9, 27], may be alleviated or negated by water level fluctuation, enabling the plant to survive under conditions of disturbance such as flooding. Furthermore, *A*. *philoxeroides* can adjust its functional traits in response to environmental changes, and this plasticity improves survival and growth probabilities for the plant. Therefore, riparian wetland plants, like *A*. *philoxeroides*, may be better able to grow and occupy wider niches in recurrently disturbed environments (e.g., areas with fluctuating water levels). Imminent increases in anthropogenic disturbance to wetland habitats, as well as changes in weather patterns, are likely to cause dramatic and rapid fluctuations in water levels, and increase the number of disturbed environments. Greater research on the adaptations and responses of plants, including invasive species like *A*. *philoxeroides*, and the comparison between native vs. invader, and between exotic non-invaders vs. invader in such frequently disturbed environments is essential to understand their invasion success, and mitigate their effects on native riparian and wetland plant communities.
